# Emphasis on heat strain to the ocular surface: A functional and clinical study of a modified goggle

**DOI:** 10.3389/fpubh.2022.955443

**Published:** 2022-08-02

**Authors:** Yuting Shao, Jingzhong Wu, Peichen Wu, Xin Liu, Jiaqi Shen, Li Zhang, Yanlong Bi

**Affiliations:** ^1^Department of Ophthalmology, School of Medicine, Tongji Hospital, Tongji University, Shanghai, China; ^2^Actif Polarizers Technology R&D Center, Xiamen, China; ^3^Department of Ophthalmology, Guizhou Provincial People's Hospital, Guiyang, China; ^4^Tongji Eye Institute, Tongji University, Shanghai, China

**Keywords:** goggles, healthcare worker (HCW), heat strain, antifog, blue light

## Abstract

**Purpose:**

The limitations of conventional goggles have caused immense inconvenience, and even damage, to the physical and mental health of healthcare workers. Hence, this study aimed to build a modified goggle (MG) with better physical performance. The temperature-humidity index (THI) was used as an indicator to investigate the impact of goggle-related heat strain on the ocular surface.

**Methods:**

The basic functions of antifog, anti-ultraviolet (UV), and anti-blue-light radiation capabilities were evaluated. Furthermore, the clinical impact on noninvasive keratography tear film break-up time (NIKBUT), intraocular pressure, central corneal thickness, Schirmer test I, and the Dry Eye-related Quality of life Score (DEQS) were assessed in 40 healthcare workers by comparing MG with standard goggles (SG). The relationships between THI and the above parameters were explored.

**Results:**

MG had a significantly longer antifog time than SG (212.75 ± 23.95 vs. 138.35 ± 5.54 min, *p* < 0.05), stronger antiultraviolet ability at 400 nm (99.99 vs. 45.55%), and optimal anti-blue-light performance at 440 nm (33.32 vs. 13.31%). Tear film stability after wearing the goggle was significantly worse than that before wearing them (*p* < 0.05). Both goggles achieved moderate to strong heat strain, with a THI of >80 at all timepoints. The MG group showed lower THI and DEQS and higher NIKBUT than the SG group (*p* < 0.05). THI was significantly correlated with DEQS, NIKBUT, and real fogging time (*r* = 0.876, −0.532, −0.406; *all p* < 0.05).

**Conclusion:**

Wearing goggles for a long time may cause heat strain to the eyes, thereby leading to eye discomfort and changes in the microenvironment of the ocular surface. Our MG exhibited better antifog, antiultraviolet, and optimal anti-blue-light performance and lower heat strain than SG, thus making it ideally suited for healthcare workers.

## Introduction

Globally, as of July 18, 2022, severe acute respiratory syndrome coronavirus 2 (SARS-CoV-2) has infected more than 559 million individuals and caused more than 6 million deaths (1). Although vaccines are available worldwide, their effectiveness needs to be further evaluated because of the continuous mutations in the virus (2). The use of personal protective equipment (PPE) remains the best method for preventing infection.

**Figure 1:**
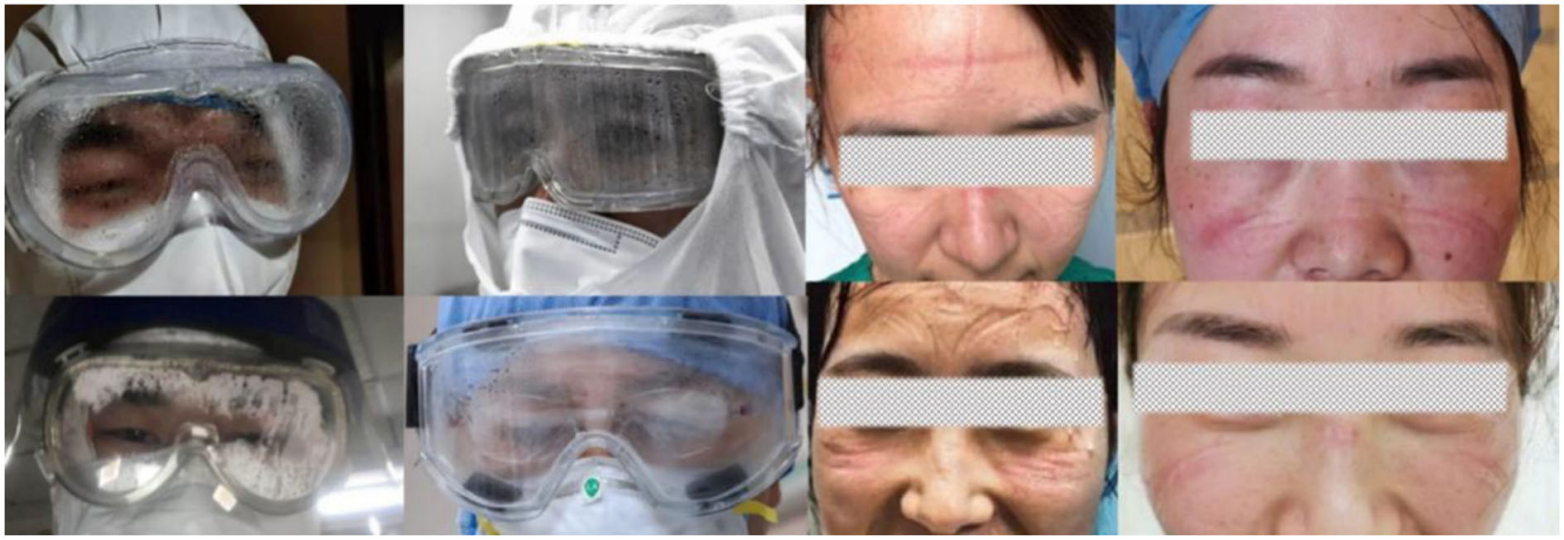
Photographs from news media reports in China indicate that goggles fogging and PPE-related pressure injuries were prominent among HCWs.

Goggles, which are eye protection devices, are of great importance in reducing the infection rate among healthcare workers (HCWs) (3). The physical barrier effect of goggles is better than that of face shields, which is especially important because evidence that COVID-19 can be transmitted through the eyes is emerging (4, 5). However, in the real world, with the sudden appearance of the epidemic, many countries have encountered a shortage of goggles and have faced problems related to their low function (6). The limitations of conventional goggles have posed immense inconvenience, and even damage, to the physical and mental health of HCWs. The goggles face issues, such as fogging (6), pressure damage (7, 8), and radiation risks (9, 10), and cause severe discomfort (11) (Figure 1). Medical goggle standards that can help in overcoming the above constraints and preventing the spread of highly infectious pathogens that expose the HCWs to potential risks are lacking.

Heat strain refers to a series of reactions in the organisms to the thermal environment. A recent article stated that many factors exist in the human thermal environment, including environmental factors (temperature, humidity, wind velocity, and solar radiation), task-dependent factors (e.g., metabolic rate and clothing), and individual factors (e.g., age, sex, body mass, morphology, and aerobic fitness). These factors cause heat strain to the cardiovascular system, central nervous system, and skeletal muscle function and result in fatigue development (12). During COVID-19, there has been a high prevalence of heat strain among HCWs because of wearing PPE, which has resulted in heat-related physical symptoms, including thirst, fatigue, sweating, uncomfortable warmth, and reduced work performance (13). However, the effect of heat strain on the eyes has not yet been studied.

Goggles cause systemic heat strain and directly affect the eyes. Previous studies have shown that brief (approximately 10 min) exposure to the high temperature (45°C−55°C)–humidity environment of goggles can effectively warm the outer eyelids and is effective against meibomian gland dysfunction (14). However, whether routine continuous (≥4 h) exposure of the eyes to the high temperature–humidity of the goggles causes heat strain and affects the function of the ocular surface and whether the subjective symptoms are related to the environment inside the goggles need to be explored.

This study aimed to create a modified goggle (MG) that has the potential to overcome the shortcomings of the standard goggles (SG) and explore the impact of heat strain on the ocular surface. A technical introduction regarding the fabrication of MG and the results of the function and cross-sectional clinical study compared with the SG from the 3M company are provided, along with a discussion based on the design considerations.

## Methods

### Study design and participants

**Figure 2:**
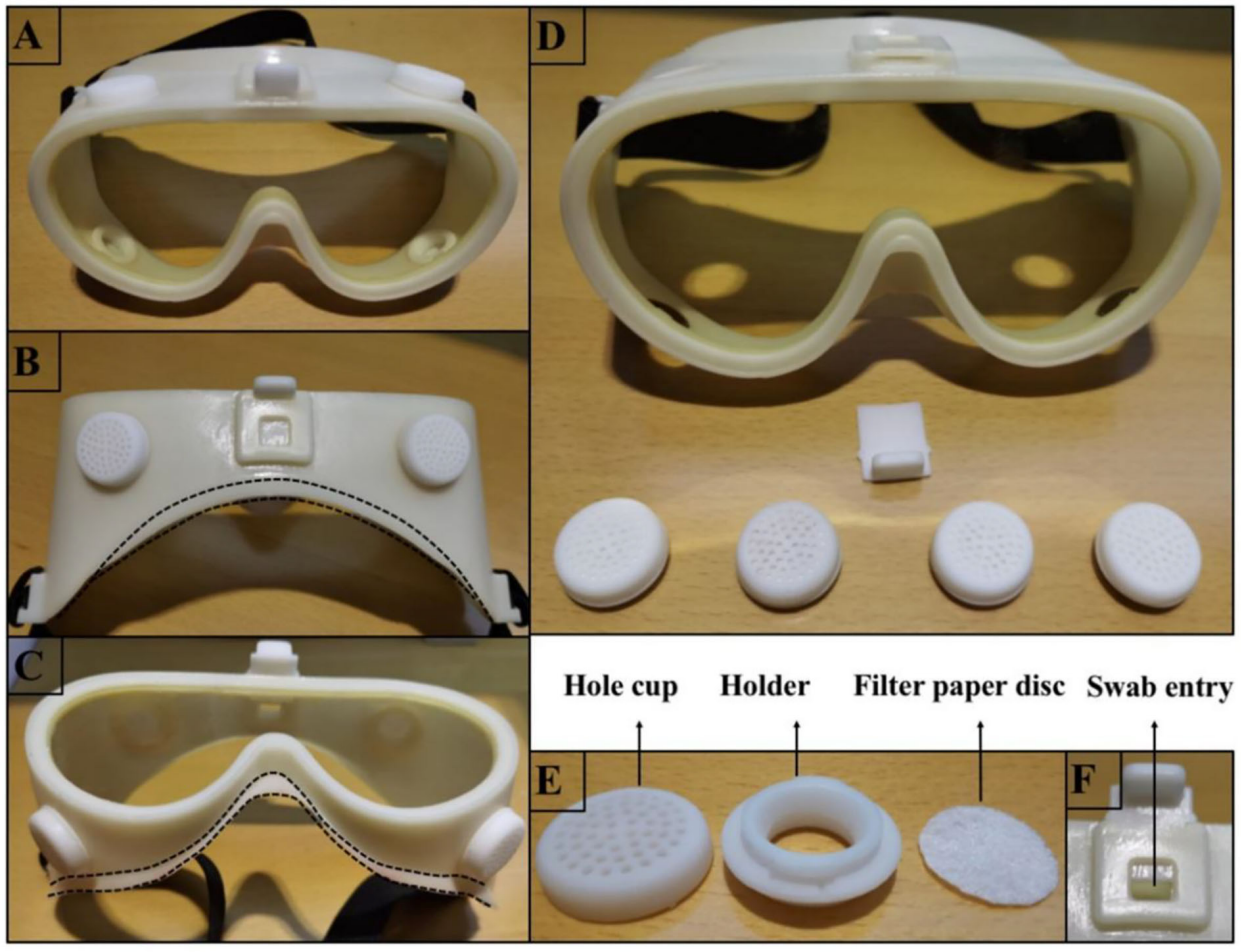
Photographs depicting how the MG was fabricated. **(A)** Shows the front view of the MG. **(B,C)** Illustrate the top and bottom views of the MG, respectively, and the dotted line indicates how the silicone portion fits the face. **(D)** Illustrates the decomposed portion of the MG, which includes 4 filters and a one-way valve. **(E)** Illustrates the decomposed portion of the filter, including the hole cup, holder, and filter-paper disc. **(F)** Illustrates the valve open state when the swab can be inserted into the goggle to fix an urgent tickle or dry the eyes without removing the goggles.

Our MG is composed of a specially designed silicone body and a lens with improved technology. The silicone body contained four virus-proof air filters and a one-way valve (Figure 2). The air filter comprised a holder, filter paper disc, and hole cup. The filter paper disc was removed from the qualified face mask. The lens provided by Actif Polarizers Technology was qualified for anti-fog, optimal anti-blue light radiation, and anti-ultraviolet radiation functions. SG (AF1621, 3M), which claims to be anti-fog and 99% anti-UV, was used to compare the performances of MG.

HCWs at Tongji Hospital affiliated with Tongji University were invited to participate in this study. Those with a completely normal ophthalmological assessment under a slit-lamp microscope (YZ5T, 66 Vision Technology, China) and related routine eye examinations were eligible to participate in the study. The exclusion criteria were a history of systemic or intraocular inflammatory diseases, including dry eye diseases, myopia with −1.00 D or lower, and systemic or topical therapies in the last 6 months that could have modified the ocular surface. Those with a history of contact lens use and myopia were asked to remove the lens or glass for a week before the test. After enrolment, the participants were assigned the goggle *via* a random number generated by “MS Excel,” with 20 participants in each group. For each subject, the fit of the goggles was carefully checked to ensure that it did not confound the results. Once the goggles were comfortable and optimally fitted, the timer was set to 240 min. The HCWs operated computers for 2 h and read books for another 2 h in one room without participating in other tasks. If the goggles were removed halfway, the subject was withdrawn from the study. The participants provided informed consent before the study, and it was approved by the Medical Ethical Committee of Tongji Hospital affiliated with Tongji University. The study adhered to the tenets of the Declaration of Helsinki.

### Function tests

The function tests included antiultraviolet, anti-blue-light, and simulated antifog capabilities before wearing. ***Antiultraviolet and anti-blue-light capability:*** The capability to resist ultraviolet and blue radiations was tested using spectral transmittance experiments that reflect the loss of light at different wavelengths through objects. Two types of goggles were tested through an independent professional agency with a UV spectrometer (SDR1911, Speedre Technology Co. Ltd, China) from 190 to 1,100 nm in 1 nm step. The goggles were positioned carefully over the entrance optics of the spectrometer. The scans were repeated at least three times, and the average value was calculated. All data were converted into line charts. ***Antifog capability:*** To evaluate the antifog capability, both simulation and real-scene tests were adopted. The simulation test was modified from GB/T31726-2015 (15). The protocol was to quickly place the two types of goggles on the mouth of a beaker containing distilled water at a temperature of 85 ± 2°C and then transfer them to the nearest printed eye chart paper after 60 s. The same region was observed within 5 s, and photographs were taken to record the degree of fogging under the same circumstance. The real-scene test was conducted during the subsequent clinical test. After wearing the goggles, the participants were requested to record the real fogging time (r-FT), which signifies the duration from no fog to the fog covering the whole lens and negatively impacting the work.

### Clinical tests

An open recruitment strategy was used, with an online link that was distributed to our professional WeChat official account of the Tongji Eye Department. Participants who met the inclusion and exclusion criteria were allowed to provide basic information, including age and sex. Clinical tests, including intraocular pressure (IOP), measured using a non-contact tonometer (NT-510, NIDEK, Japan), central corneal thickness (CCT) using an anterior segment optical correlation tomography (Visante^TM^, ZEISS, Germany), median noninvasive keratography tear film break-up time (NIKBUT) using a Keratograph (5M, OCULUS, German), and Schirmer test I (STI) using a tear filter strip (Jingming, China), were performed in the right eyes of the 40 participants before and after wearing the random goggles for 4 h. IOP, CCT, and NIKBUT were repeated three times, and the average value was calculated. All participants completed the test in the same working environment (temperature, 27°C; relative humidity, 50%) and performed the same activities. Data on the subjective symptoms were collected using the Dry Eye-related Quality of life Score (DEQS) questionnaire, with 15 questions every 30 min at 30, 60, 90, 120, 150, 180, 210, and 240 min. The results were used to assess not only the degree of bothersome eye symptoms but also the impact on daily life (16). The score for each item ranged from 0 to 4 points. The higher the score, the more serious are the symptoms. Temperature and relative humidity inside the goggles were recorded during the test using a mini temperature–humidity calculator (ABS-8845, DELI, China) for 30 min, along with the DEQS questionnaire. The temperature–humidity index (THI), which combines temperature and humidity as a single value, was calculated using the following formula (17):


$$\begin{array}{l} \text{THI}=(\text{1}.\text{8}\times \text{T+32})-[(\text{0}.\text{55}-\text{0}.\text{0055 }\times \text{ RH})\\ \text{ + }(\text{1}.\text{8 }\times \text{ T}-\text{26})], \end{array}$$

The correlations between THI and the above parameters were further studied.

### Statistical analyses

Statistical analyses were performed using the SPSS software (version 20.0; IBM, Armonk, NY, USA). The data were checked for normality using the Shapiro–Wilk test. Means with standard deviations (SD) or medians [interquartile ranges] were used to record the descriptive variables, whereas counts and percentages were used for categorical variables. The age and sex differences between the two groups were calculated using an independent sample *t*-test and chi-square test, respectively. One-way repeat measures ANOVA was used to compare the differences in THI and DEQS between the MG and SG groups at all timepoints. Differences in IOP, CCT, NIKBUT, and STI between and within groups were evaluated using an independent sample *t*-test or paired *t*-test. The r-FT between the two groups was compared using an independent sample *t*-test. Pearson or Spearman correlation coefficients were used to determine the correlation between THI, temperature, and humidity and DEQS, IOP, CCT, NIKBUT, STI, and r-FT. Statistical significance was set at *p* < 0.05.

## Results

### Antifog test

**Figure 3:**
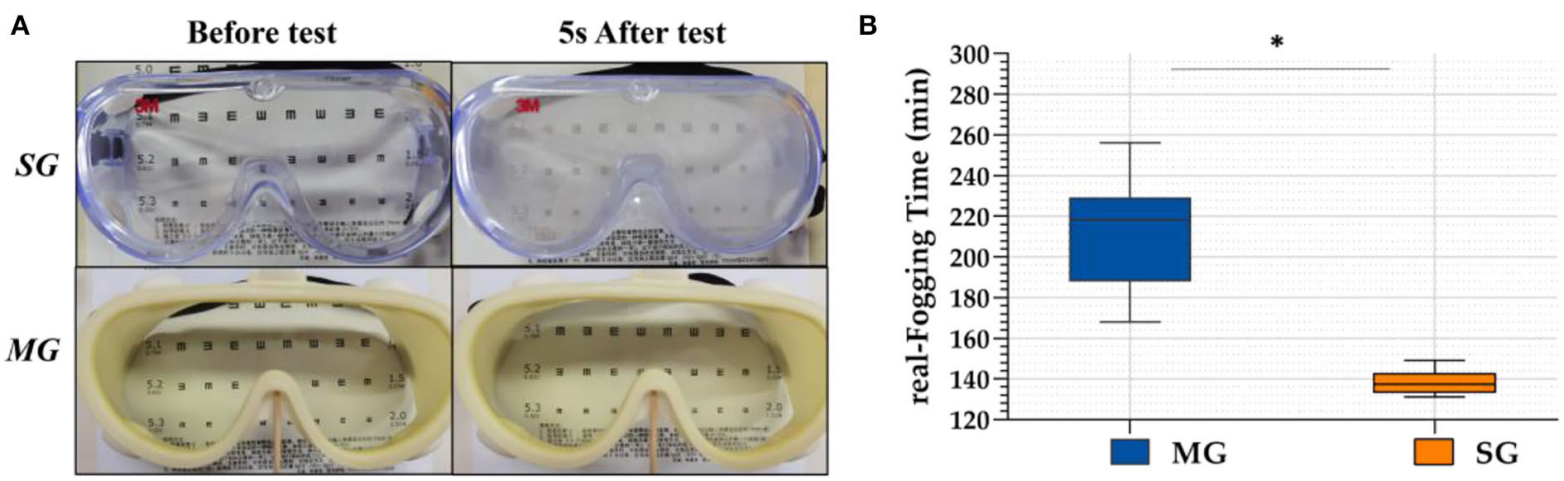
Antifog capability in simulated test **(A)** and real scene test **(B)**. *p < 0.05.

In the simulation test, when the two kinds of goggles were placed on the cup where the hot water vapor overflowed, the SG fogged immediately and the eye chart became blurred and illegible, whereas the chart under the MG was clearly visible, as shown in Figure 3. In the real scene test, r-FTs of the 40 HCWs wearing SG and MG were 138.35 ± 5.54 min and 212.75 ± 23.95 min, respectively. Statistically significant differences were observed between the SG and MG (*t* = 5.497, *p* < 0.05) groups.

### Ultraviolet and blue-light transmittance test

**Figure 4:**
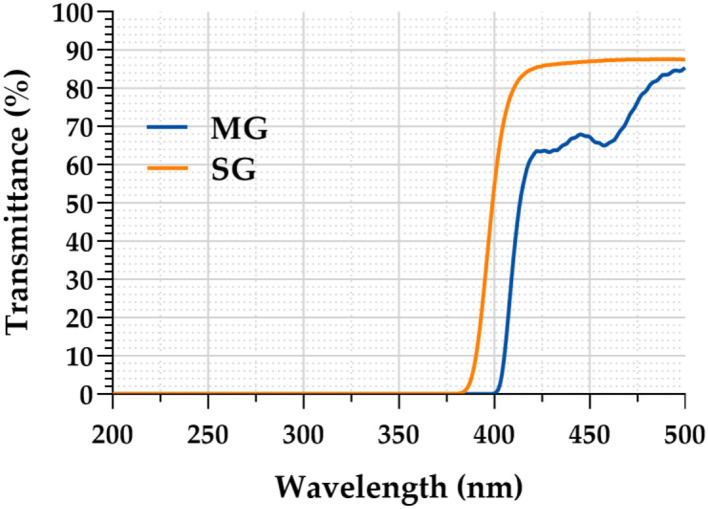
Anti-ultraviolet and blue-light capability in light transmittance test.

As clearly demonstrated in Figure 4, the light transmittance of the MG was only 0.1% in the ultraviolet band (200–400 nm), whereas that of the SG increased rapidly after 365 nm, reaching 54.45% at 400 nm. The light transmittance of the MG in the blue light band (400–500 nm) was significantly lower than that of the SG. MG reached 66.68% at 440 nm, whereas SG reached 86.69% at the same wavelength.

### Subject characteristics

The mean age of the MG group was 28.65 ± 3.54 years and that of the SG group was 29.90 ± 3.68 years. There were eight men participants (40.00%) in the MG group and seven (35.00%) in the SG group. There were no significant differences between the two groups (*t* = −1.094, *p* = 0.281; χ^2^ = 0.107, *p* = 0.744).

### Ocular surface index

The four parameters were recorded before and after the test and analyzed, as shown in Table 1. In both the MG and SG groups, the m-NIKBUT was significantly lower than the baseline (t = 6.516, t = 9.463; both *p* < 0.05). The STI was significantly higher than the baseline (t = −3.416, *p* < 0.05) in the SG group but not in the MG group (t = −1.360, *p* = 0.182). There were no differences in any of the parameters between the groups before the test. After the test, no differences were observed in IOP, CCT, and STI between the groups, but a difference was seen in NIKBUT (t = 5.172, *p* < 0.05).

**Table 1:** Ocular surface index before and after tests between MG group and SG group.

**Group**		**IOP (mmHg)**	**CCT (um)**	**NIKBUT (s)**	**STI (mm)**
MG	Before	15.24 ± 1.69	523.60 ± 13.67	12.86 ± 1.07	13.94 ± 2.34
	After	14.70 ± 1.89	531.30 ± 13.31	10.54 ± 1.18*	14.84 ± 1.81
SG	Before	15.22 ± 1.60	529.50 ± 21.09	13.44 ± 1.92	13.27 ± 2.12
	After	14.86 ± 1.86	530.95 ± 21.99	8.43 ± 1.38*	15.41 ± 1.83*
After-*p* value		0.789	0.952	<0.05	0.324

*The parameter was significantly different from the baseline.

*IOP is short for intraocular pressure; CCT is short for central corneal thickness, NIKBUT is short for noninvasive keratography tear film break-up time; STI is short for Schirmer test I*.

### Dry eye-related quality of life score

A total of 320 questionnaires were obtained from the 40 HCWs. DEQS values increased with the increase in goggle wearing time (MG-*F* = 127.91, *p* < 0.05; SG-*F* = 81.23, *p* < 0.05). Goggle types and timepoints had no interaction (*F* = 2.245, *p* = 0.11). The MG group attained significantly lower DEQS values at all timepoints than the SG group (*F* = 31.05, *p* < 0.05).

The results of the DEQS completed by all the participants at 240 min are shown in Table 2. The items of painful or sore eyes, ocular fatigue, blurred vision when watching something, problems with eyes when reading, problems with eyes when watching television or looking at a computer or cell phone, and eye symptoms affect work showed relatively high scores in both groups. Furthermore, painful or sore eyes, ocular fatigue, problems with eyes when watching television or looking at a computer or cell phone, feeling distracted because of eye symptoms, and eye symptoms affect work showed significant differences between the MG and SG groups (*all p* < 0.05).

**Table 2:** Results of DEQS items at 240 min-timepoint analysis in MG and SG groups.

**Item**	**MG**		**SG**	
	**Average**	**Proportion**	**Average**	**Proportion**
**Bothersome ocular symptoms**				
Foreign body sensation	1.0	4.72%	1.0	3.65%
Dry sensation in eyes	0.3	1.42%	0.3	1.15%
*Painful or sore eyes	2.2	10.38%	2.7	10.36%
*Ocular fatigue	1.9	8.73%	3.1	11.90%
Heavy sensation in eyelids	1.2	5.66%	1.3	4.99%
Redness in eyes	0.3	1.42%	0.2	1.92%
**Impact on daily life**				
Difficulty opening eyes	0.8	3.54%	0.3	4.80%
Blurred vision when watching something	2.3	10.61%	2.5	9.40%
Sensitivity to bright light	1.5	7.08%	1.4	5.18%
Problems with eyes when reading	2.3	10.61%	2.6	9.79%
*Problems with eyes when watching television or looking at a computer or cell phone	1.9	8.73%	2.6	9.79%
*Feeling distracted because of eye symptoms	1.7	8.02%	2.3	8.64%
*Eye symptoms affect work	1.8	8.49%	2.7	10.17%
Not feeling like going out because of eye symptoms	1.1	5.19%	0.6	2.30%
Feeling depressed because of eye symptoms	1.2	5.42%	1.6	5.95%

*The corresponding parameters showed statistical differences between groups.

### Temperature–humidity index and correlations

**Figure 5:**
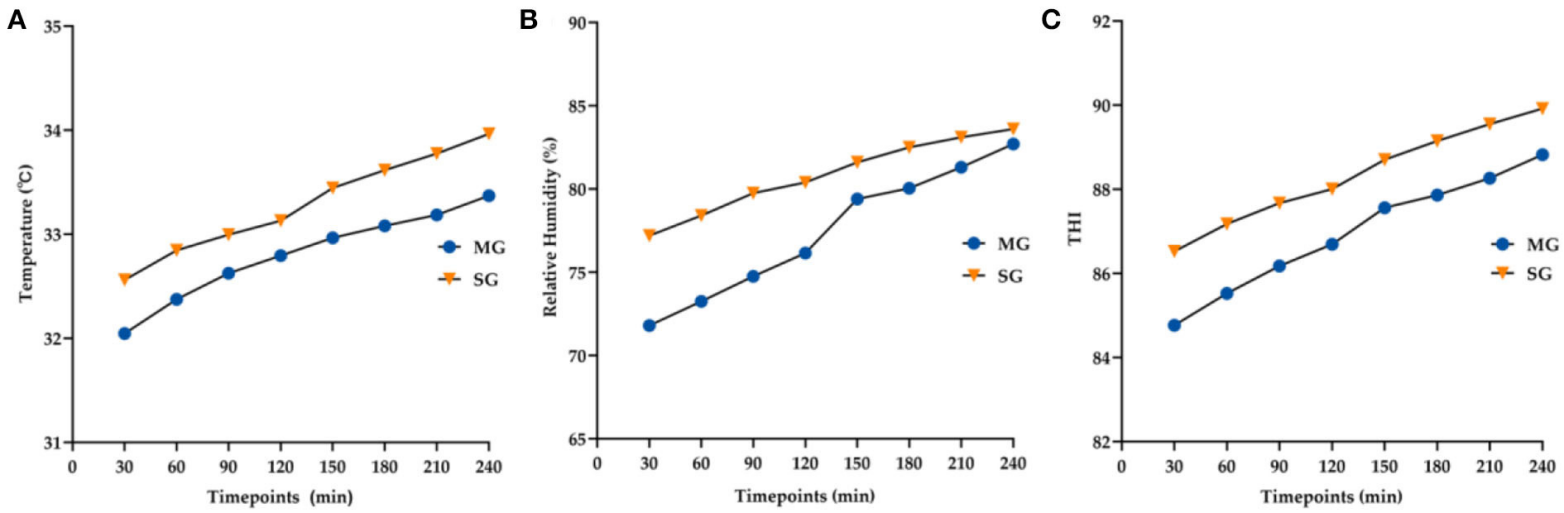
Changes in the average temperature **(A)**, relative humidity **(B)**, and THI **(C)** at all time points between the two groups.

The average temperature, relative humidity, and THI of the two groups at each timepoint are shown in Figure 5. THI values increased with the increase in goggle-wearing time (MG-*F* = 179.95, *p* < 0.05; SG-*F* = 123.32, *p* < 0.05) and were >80 at all timepoints. Goggle types and timepoints had no interaction (*F* = 2.20, *p* = 0.129). The MG group attained significantly lower THI at all timepoints than the SG group (*F* = 21.07, *p* < 0.05).

The relationships and coefficients among THI, temperature, humidity and the above parameters are described in Table 3. The results demonstrated that THI had a stronger correlation with DEQS than temperature or humidity. THI was also negatively related to NIKBUT and r-FT.

**Table 3:** Relationships and coefficients between the parameters and THI, temperature, humidity.

**Coefficients**	**THI**	**Temperature**	**Humidity**
DEQS	0.876*	0.740*	0.807*
IOP	−0.218	−0.221	0.119
CCT	0.161	0.223	−0.094
NIKBUT	–0.532*	–0.461*	−0.267
STI	0.276	0.271	−0.069
r-FT	–0.406*	–0.368*	−0.204

*The corresponding parameters showed significant statistical relationships.

## Discussion

Increasing evidence supports the possibility of virus transmission through the ocular surface (4, 5). Therefore, the use of goggles as a physical barrier is recommended for HCWs. However, there are no unified medical goggle standards at home or abroad. Low functional goggles have caused great trouble and resulted in injury to HCWs during the epidemic and have also been responsible for more potentially unknown damage. The recommended performances of medical goggles have been reviewed in our previous study (18).

**Figure 6:**
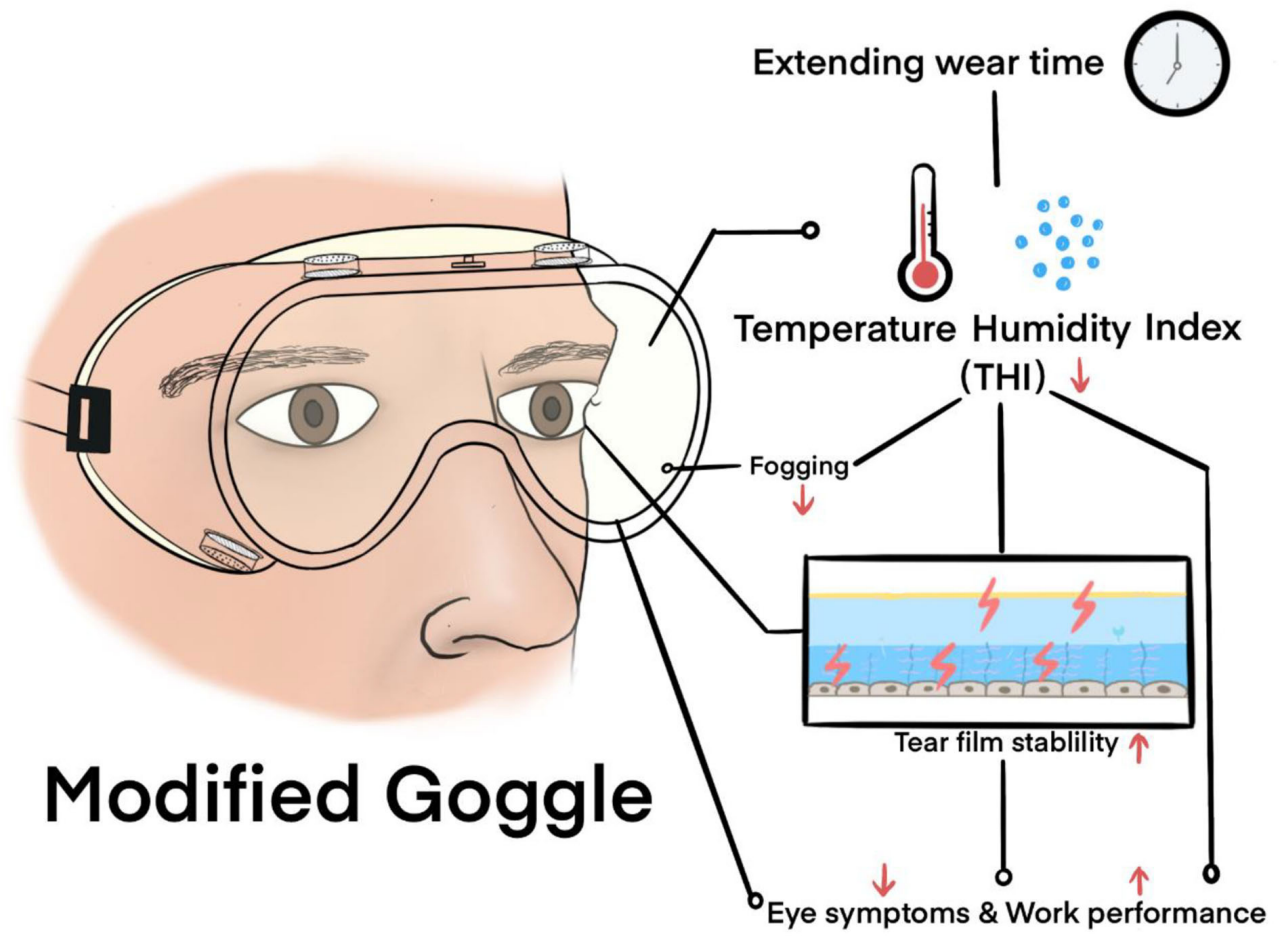
The possible clinical mechanisms of heat strain to the eye and preferable effect of MG.

In this study, an MG with antifog, antiultraviolet, and optimal anti-blue-light radiation capabilities was introduced and evaluated *via* function and clinical tests. To the best of our knowledge, this is the first study to propose that goggle-related heat strain acts on the ocular surface (Figure 6). The standard antifog and antiultraviolet goggles manufactured by the 3M company were selected as a control, which makes the research convincing and representative.

In the function tests, the r-FT of MG was 212.75 ± 23.95 min, which was 1.5 times longer than that of SG, mainly due to the specific manufacturing process of the lenses and the optimized main body design that lower both temperature and humidity. Kumar et al. stated that detergent-based surfactant is a low-cost technique that controls the fogging of goggles and provides a longer duration of clear visibility (69.3 ± 8.16 min) than antifog polyethylene terephthalate films and filtered vents (19). However, detergent-based surfactants cause frequent eye irritation and slightly distort the vision if the soap is not properly wiped (20). Bhardwaj et al. opined that a simple solution to the fogging problems is to avoid airflow *via* the application of Micropore^TM^ or other paper-based adhesive tapes to the upper margin of the mask and the skin (21). However, all these methods have limited antifog effects and involve complicated steps. Our research proved that not only should the antifog capacity of the lens be optimized but also the temperature and humidity inside the goggles must be simultaneously reduced.

It is well known that wearing goggles for a short time (no more than 20 min) can increase the humidity around the eyelids, thereby reducing the symptoms of dry eye disease and meibomian gland dysfunction (14, 22). However, whether exposure to relatively high humidity and temperature for a long time (more than 4 h, the normal shift time) affects the ocular surface has not yet been examined. Research has shown that extended exposure to temperatures >25°C along with high humidity can cause heat strain to the human body, especially the cardiovascular, central nervous, and skeletal muscle systems, and result in fatigue development (12, 17). Environmental factors (temperature, humidity, wind velocity, and solar radiation), task-dependent factors (e.g., metabolic rate and clothing), and individual factors (e.g., age, sex, body mass, morphology, and aerobic fitness) are involved in heat strain (12). Air movement inside the goggles is low, the indoor solar radiation is effectively blocked, and the work intensity and clothing were controlled by the test. Hence, THI (a formula involving only temperature and humidity) was selected to measure the heat strain inside the goggles rather than other usual indicators, such as the universal thermal climate index and predicted heat strain. The THI was initially developed to quantify the discomfort felt by a human during the summer and was later extended to estimate the heat stress on livestock (23, 24). Unexpectedly, it was found that during the 4 h of wearing the goggles, the eyes were exposed to a moderate to strong heat strain (80 ≤ THI ≤ 89) at all time points and that THI increased gradually with the extension of wearing time to a very strong strain (>89).

The DEQS was used to evaluate the subjective symptoms of the participants. Although the ocular surface disease index is widely used to diagnose and evaluate the symptom severity, it does not fully cover the effect on the lives of the subjects (16). Therefore, the DEQS questionnaire was selected. Bongers et al. observed that HCWs experienced approximately 25 times greater heat strain symptoms while performing medical duties with PPE (93% of HCWs) than that without PPE (30% HCWs) (13). The reported heat strain symptoms include thirst, fatigue, (excessive) sweating, and uncomfortable warmth. Their effects are slower work performance and less accurate execution of work activities (25). According to the results of our questionnaire, painful or sore eyes and ocular fatigue were the main eye symptoms. Others included the impact of blurred vision when watching something, problems with eyes when reading, problems with eyes when watching television or looking at a computer or cell phone, and eye symptoms affecting work. Our study revealed that the alterations in DEQS were significantly related to THI, with an *r*_*p*_ value of 0.876. This finding suggests that heat strain causes eye and eye-related systemic symptoms. The MG group showed significantly lower DEQS and THI than the SG group at all time points, which is indicative of a superior wearing experience.

To further study the effect of heat strain on ocular surface function, the ocular surfaces of the 40 participants who wore goggles for four consecutive hours were clinically evaluated. IOP and CCTs did not reveal any significant intergroup differences. However, a significant decrease in NIKBUT occurred in each group, which agrees with the results of Vera et al. (26). Tear film breakup results from the linear thinning of the tear film between blinks, which may be due to the flow of tears in three directions: outward (i.e., evaporation), the inward flow of water into the corneal epithelium, and tangential flow along the surface of the epithelium (27). The normal reference value for human central corneal temperature is 32.6 ± 0.70°C (28). In our study, the temperature inside the goggles exceeded the critical value after wearing them for 30–60 min, which might have led to the evaporation of tears on the ocular surface and the instability of the tear film. Correlation analysis further confirmed that NIKBUT showed a significant negative correlation with THI and temperature. Although there was no significant correlation between high humidity (>70%) and the decrease in NIKBUT, the following direct or indirect evidence revealed that appropriate humidity was necessary to tear film stability. First, a previous study demonstrated that the humidity decreases by 5% in 1 h when the temperature remains unchanged and that the tear film rupture time is significantly shortened (29). Second, the tear film remained stable when the temperature exceeded this value in summer and the humidity was 30–60%. In addition, the STI was found to increase significantly in only the SG group, but no significant correlation with THI was seen. Hence, it was speculated that ocular fatigue caused by heat strain or pungent odor resulted in lacrimal reflex secretion. The sample size needs to be further increased. In general, the results showed that wearing the goggles for 4 h affected the microenvironment of the ocular surface. The MG showed a better subjective feeling, which could be attributed to fewer changes in ocular surface functions caused by the heat strain.

The pressure on the face also affects comfort and is highly related to the material of the skin-contact surface. The silicone rubber, which is more skin-friendly and has a lower coefficient of friction than polyethylene, was used (30). The one-way valve that allows the HCWs to use a sterile cotton swab to tickle without removing the goggles is an important humanized design.

Finally, ultraviolet and blue light are often overlooked in medical settings. The use of ultraviolet light for disinfection is quite common and harms the eye and the surrounding skin (8–10). Many electronic devices transmit blue light (420–460 nm), which harms the oculus (31). Compared with the SG, the MG exhibited a stronger blocking performance of ultraviolet and blue-light radiation, thereby preventing potential damage to the eyes. Our previous study confirmed that shielding approximately 30% of the blue light can improve the accommodation of the human eye and ocular fatigue (32, 33). The data from the MG group showed that the scores for painful or sore eyes, ocular fatigue, problems with eyes when watching television or looking at a computer or cell phone, and eye symptoms affecting work were significantly lower than those of the SG group, which could also be attributed to the optimal anti-blue-light performance of the lens.

In conclusion, the MG showed improvements in several aspects compared with the SG, but some problems still exist. For example, (I) the antivirus capability is hard to test; (II) the main body of the goggles should be transparent to expand the field of view; (III) lack of objective examinations, such as *in vivo* confocal microscopy, to detect the morphologic changes in all kinds of cells under heat strain; (IV) in the real environment, the shortage of medical staff increases the continuous working hours and the activity associated with heavy tasks, thus leading to a greater degree of heat strain to the eye than that reported in this study; (V) the humidity and temperature inside the goggle need to be decreased further to relieve heat strain; (VI) the molecular mechanism of heat strain on the ocular surface needs further elucidation.

The findings from this study establish that the MG exhibits better antifog, antiultraviolet, and optimal anti-blue-light performance and lower heat strain than the SG. The heat strain to the eyes of HCWs caused by the wearing of goggles for a long time in medical settings cannot be ignored. Optimizing the goggles and formulating medical goggle standards are, hence, required.

## Data availability statement

The raw data supporting the conclusions of this article will be made available by the authors, without undue reservation.

## Ethics statement

The studies involving human participants were reviewed and approved by the Medical Ethical Committee of Tongji Hospital affiliated to Tongji University. The patients/participants provided their written informed consent to participate in this study.

## Author contributions

This work is the result of collaboration with JW and PW. YB and LZ: conception and review. JS and XL: clinical operation. YS: methodology, formal analysis, and writing. YB: funding acquisition. All authors contributed to the article and approved the submitted version.

## Funding

This work was funded by Study on Multifunctional Medical Goggles in COVID-19, (Grant No. PA2020000088), Development and Application of Animal Model of Corneal Epithelial Cell Disease, (Grant No. 201409006500), and Major Clinical Research Projects of the Three-Year Action Plan for Promoting Clinical Skills and Clinical Innovation in Municipal Hospitals, (Grant No. SHDC2020CR1043B-010).

## Conflict of interest

The authors declare that the research was conducted in the absence of any commercial or financial relationships that could be construed as a potential conflict of interest.

## Publisher's note

All claims expressed in this article are solely those of the authors and do not necessarily represent those of their affiliated organizations, or those of the publisher, the editors and the reviewers. Any product that may be evaluated in this article, or claim that may be made by its manufacturer, is not guaranteed or endorsed by the publisher.
